# Naturally-primed life strategy plasticity of dimorphic *Aethionema arabicum* facilitates optimal habitat colonization

**DOI:** 10.1038/s41598-019-52520-y

**Published:** 2019-11-06

**Authors:** Samik Bhattacharya, Katja Sperber, Barış Özüdoğru, Gerhard Leubner-Metzger, Klaus Mummenhoff

**Affiliations:** 1 Department of Biology, Botany, University of Osnabrück, Barbarastraße 11, D-49076 Osnabrück, Germany; 2 Department of Biology, Faculty of Science, Hacettepe University, Beytepe, Ankara 06800 Turkey; 3 School of Biological Sciences, Royal Holloway University of London, Egham, Surrey TW20 0EX United Kingdom; 4 Laboratory of Growth Regulators, Centre of the Region Haná for Biotechnological and Agricultural Research, Palacký University and Institute of Experimental Botany, Academy of Sciences of the Czech Republic, 78371 Olomouc, Czech Republic

**Keywords:** Plant morphogenesis, Plant ecology, Plant stress responses

## Abstract

Plasticity in plant dispersal traits can maximise the ability of a plant species to survive in stressful environments during colonization. *Aethionema arabicum* (Brassicaceae) is a dimorphic annual species that is hypothesized to survive stressful conditions during colonization due to adaptive plasticity in life-phase (vegetative vs sexual) and fruit morph (dehiscent [DEH] vs indehiscent fruits [IND]). We tested for adaptive plasticity in life-phase and fruit morphs along laboratory environmental stress gradients found in the natural habitats of *Ae. arabicum*. We considered optimal environmental conditions (750–2000 m above sea level) to be those that resulted in the following fitness parameters: higher biomass and a higher total number of fruits compared to stressful habitats. We found evidence of plasticity in life-phase and fruit-morph along a stressful environmental gradient. High hydrothermal stress proportionally increased the number of dehiscent morphs and non-dormant seeds germinating in autumn. This offsets natural phenology towards dry and cold winter (less hydrothermal stress), yielding fewer fruits that dehisce in the next generation. We conclude that the plastic responses of *Ae. arabicum* to natural stress gradients constitute a strategy of long-term adaptive benefits and favouring potential pathways of colonisation of the optimal habitat.

## Introduction

Spatial distribution of species diversity is vital to our understanding of why species assembly within the same area tends to be distinct from assemblages anywhere else^1^, as introduced by Darwin^2^ and Wallace^3^ in their seminal work on biogeography. While animal migration to find suitable habitats is distinctly visible, for sessile plants, the strategy is cryptic and mostly involves adaptive structural/functional modifications suitable for such colonization. Morpho-physiologically diverse fruits and seeds (heteromorphic diaspores) and phenotypic plasticity are thought to influence greatly population establishment and local adaptation^4,5^. Moreover, plasticity plays an important role during environmental change or colonization and is likely to favour plastic individuals to more successfully survive and reproduce than the less plastic individuals^6,7^. However, empirical evidence is scarce on whether plants can tune their life strategies to colonize optimal habitat^8,9^.

The production of distinct heteromorphic diaspores is considered as an outcome of constant fine-tuning of different tactics for successful dispersal and appropriate germination timing^10,11^. This diversity allows alternative strategies of dispersal, germination, dormancy, and seedling competitive ability^12,13^. Both spatial dispersal and dormancy are considered part of ‘bet-hedging’ strategies in unpredictable environments, where offspring are distributed over space or time to optimize their opportunity for successful reproduction irrespective of the prevailing conditions. This strategy maximizes the geometric mean fitness across generations, which is very sensitive to extreme environmental conditions^14^, as on mountain slopes.

Early eco-physiological perspectives for classification of plant species^15^ considered two apparently independent environmental variables, stress and disturbance, to propose that inhabitants within continuously unproductive, disturbed conditions (e.g., cold and warm deserts, mountains) exhibit a stress-tolerant strategy^16^. Organisms living at the boundaries (edges) of their ecological niches may experience environmental conditions that deviate from the optimal conditions for normal growth and development^17,18^. The induction of phenotypic plasticity via environmental cues are well documented (reviewed by Morris^7^, and references therein), however, a thorough understanding is lacking about the plastic adaptation to changing environment at different altitudinal ranges and calls for more integrative eco-physiological studies.

From an adaptive viewpoint, fruit and seed heteromorphism might pose a predicament, because not all diaspores are optimally adapted to the prevalent environmental conditions, which risk the long-term reproductive success of the offspring. Whereas an ecological perspective highlights the advantages of diverse fruit morphs considering their development is often plastically synchronized to abiotic^19–21^ and biotic^13^ stress experienced by the mother plant at different ontogenetic stages. As in the heteromorphic *Diptychocarpus strictus* (Brassicaceae), both severe water stress and simulated herbivory by defoliation^22^ led to the production of a greater proportion of indehiscent fruits. The diverse morphology of heteromorphic species, living in arid and semi-arid, stressful habitats^23,24^, provides an excellent opportunity for the diaspores to escape uncertain fate in time and space from the complex environmental gradient in the mountainous terrain.

**Figure 1:**
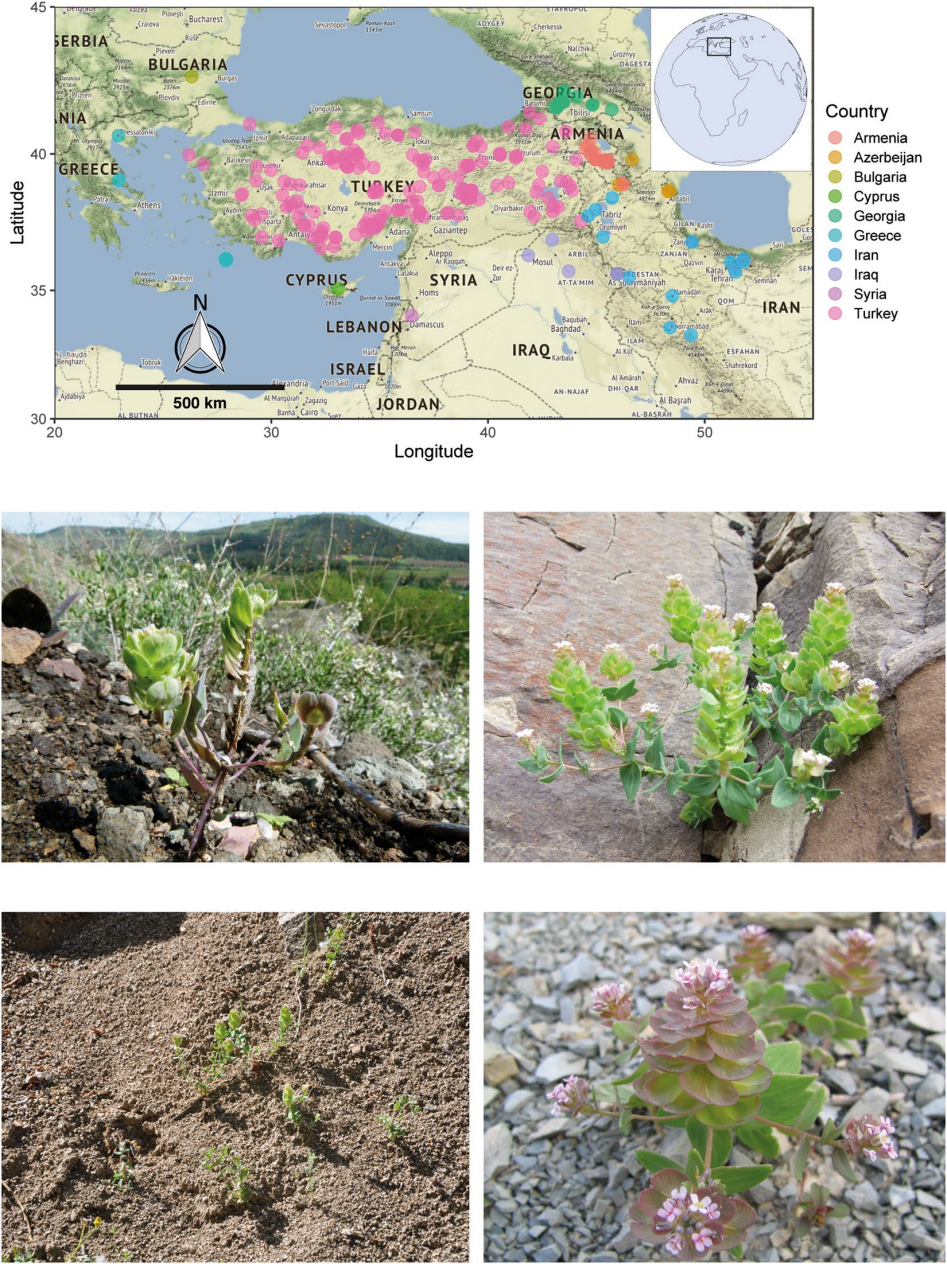
The bio-geographic diversity of natural habitats of *Aethionema arabicum* accessions in ten East Mediterranean/Irano-Turanian countries. The coloured circles represent the common sites of occurrence of *Ae. arabicum*. Representative pictures highlight the natural stress gradient in habitats of *Ae. arabicum* in scree, stony and mountainous slopes with low inter- and intra-specific competition. Harsh climatic and edaphic conditions at the natural habitat are evident in the arid and semi-arid environments of the Irano-Turanian region. Picture courtesy: Bariş Özüdoğru (Hacettepe University, Turkey), Kurtuluş Özgişi, Osmangazi University, Eskişehir. The map was generated using the software R (ver. 3.4.3) and package ‘ggmap’.

*Aethionema arabicum* (L.) Andrz. ex DC. is an annual member of the Aethionemeae and occurs mainly in the East Mediterranean and Irano-Turanian region^25^, the hypothetical centre of origin for Brassicaceae^26,27^. The species constantly experiences unpredictable climatic conditions in the semi-arid steppe (Central Anatolian Plateau), along elevations of the mountain slopes and screes (Fig. 1) with occasional microclimates along elevation in the major mountain belts^28^. *Aethionema arabicum* exhibits true dimorphism of the fruit (e.g., size, seed number, septum formation, fruit dehiscence) and seeds (e.g., surface structure, mucilage production, and germination behaviour) with complete lack of intermediate morph^29,30^. While the morph with low dispersal ability remain close to mother plants and germinate more quickly (mucilaginous seeds released from dehiscent fruits, DEH), the morph with high dispersal ability germinate later (indehiscent fruits, IND) due to their dormancy^31^. Such a combination has also been observed in other species and interpreted as a way to overcome high sibling competition in local populations^14,32^. The dimorphism displayed dynamic flexibility in the production of reproductive parts^33^ and in the proportion of two fruit morphs (DEH: IND) produced on the same plant in response to temperature^29^, branch removal^33,34^, mechanical damage, and herbivory^35^. This distinct dimorphism of *Ae. arabicum* presents a fascinating model system with which the optimal plastic response of parental generation is observed that allows the plants to modulate their dispersal and germination ability in response to environmental cues thereby gaining strategic advantage potentially via bet-hedging.

Based on the above premise, we hypothesized that the environmental gradients along the habitats of *Ae. arabicum* regulate plasticity in life phases (vegetative and sexual) and fruit morphs (DEH and IND) which promotes colonisation at the optimal habitat. In this study, we used an integrative empirical and meta-analytical approach i) to estimate the plant fitness and plasticity of life phases and fruit morphs in response to the natural equivalent of optimal (as control) and sub-optimal (as stress) abiotic and biotic conditions, and ii) to interpret the consequence of stress-mediated plasticity upon the life-strategies of dimorphic *Ae. arabicum* and possible pathways of migration towards its optimal habitat.

## Results and Discussion

### Distinct environmental and edaphic factors define optimal habitat of *Aethionema arabicum*

#### Environmental factors

An extensive survey of 238 *Ae. arabicum* collection sites in 10 countries on the East Mediterranean/Irano-Turanian mountain slopes highlighted the unpredictable and stressful natural habitats of the species (Fig. 1). Detailed analysis of climatic and edaphic factors along the elevations (Materials and Methods, Table S1) revealed a distinct ‘optimal habitat’ between an elevation of 750–2000 meters above sea level (masl) that supports the natural phenology of the species (Fig. S1a).

During germination of seeds and seedling establishment in spring, the monthly mean temperature at the optimal habitat remains 14 ± 2 °C (with day/night variation of 18/12 °C), while during vegetative and reproductive growth the summer temperature is 20 ± 2 °C. However, the summer temperature is higher than optimal (24 °C, T_max_ = 33 °C) at the lower edge of the optimal habitat (0–750 masl). On the other hand, the spring temperature at the upper edge of the optimal habitat (2000–3000 masl) is sub-optimal (3 ± 2 °C, T_min_ = −1.2 °C, Table S1) for germination of seeds and seedling establishment but suitable for vegetative and reproductive growth during summer (15 ± 1 °C). Rainfall was consistently scarcer during the summer months (17 ± 4 mm), irrespective of the elevation along the mountain slope (Fig. S1b) than in autumn (36 ± 8 mm), spring (57 ± 9 mm) and winter (55 ± 20 mm), which is typical of Mediterranean/Irano-Turanian climate.

The congregation of *Ae. arabicum* plants between 750–2000 masl on the rocky mountain slopes and screes (Figs 1, S1), defined from an exhaustive biogeographical survey and curve fitting of environmental parameters, falls within the ‘Irano-Turanian bioclimate’, with characteristic hot and dry summers and cold and moist winters^36^. Field observations in the Central and East Anatolian region (Çağatay Tavşanoğlu, Hacettepe University, Ankara, Turkey; M. Firat, Yüzüncüyıl University, Van, Turkey, Kurtuluş Özgişi, Osmangazi University, Eskişehir) confirmed the abundance of *Ae. arabicum* at the optimal habitat. While the basic geophysical parameters driving plant adaptation along the mountain slope is often studied in tandem with regional parameters^37,38^, the surface topology may confound such parameters and differs strongly from standard meteorological data^39^. Therefore, we considered all interacting factors equally during our study and analysed their overall effect on phenotypic plasticity.

#### Edaphic factors

The observed occurrence of *Ae. arabicum* on the stony mountain slopes and screes (Fig. 1) was consistent with their aggregation in habitats showing a more coarse soil profile above 750 masl than at the lower elevations (Fig. S1b). Although soil organic carbon and cation exchange capacity varied marginally with altitude (Fig. S1b), an expected decease in nutrient availability at high altitude on the Irano-Turanian mountain slopes^40^ prompted us to test three progressively declining nutrient availability grades. Interestingly, soil coarseness and other edaphic conditions also aggravate the scarcity of water at sub-alpine and alpine altitudes (>2000 masl) with low precipitation, which physiologically delimits the occurrence of cohabitants due to drought and nutrient stress^41^. Therefore, we tested the relevant intra-specific competitive stress gradient on plasticity.

### Control parameters of the optimal habitat provide ideal fitness to *Aethionema arabicum*

**Figure 2:**
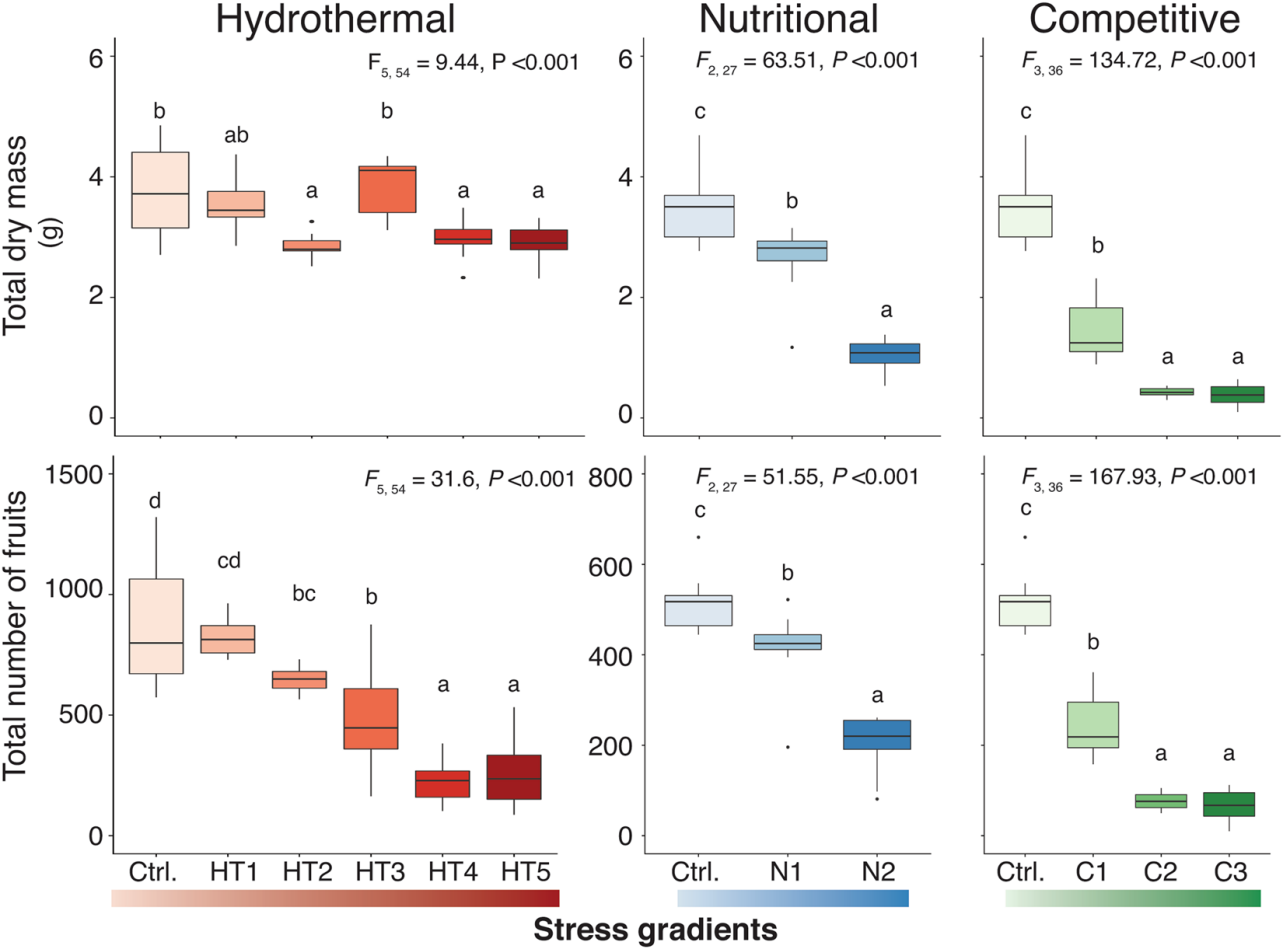
Higher fitness of *Aethionema arabicum* (total dry mass and total number of fruits produced in a lifetime) was achieved in experimental simulation of abiotic and biotic conditions of the optimal habitat than of the sub-optimal and stressful lower and upper habitats. Total biomass (g, vegetative and reproductive parts) and total number of fruits (dehiscent and indehiscent) produced in a lifetime per plant were represented as box and whisker plots. The box delimits the first and third quartiles of the data; the solid line within the box represents the second quartile, whiskers, upper and lower fence; and dots, outliers. Different letters (a, b, c, and d) designate significantly different means as determined by Tukey’s HSD (P < 0.001) of an ANOVA. Control experiments (Ctrl., ca. 55 mm rainfall, 20/12 °C, day/night), hydrothermal stress gradients (HT1, ca. 35 mm rainfall, 20/12 °C; HT2, ca. 20 mm rainfall, 20/12 °C; HT3, ca. 55 mm rainfall, 25/20 °C; HT4, 35 mm, 25/20 °C; HT5, 20 mm, 25/20 °C), nutritional stress gradients (N1, 0,1%; N2, 0% v/v fertilizer), and competitive stress gradients (C1, 3 plants, C2, 5 plants, C3, 7 plants per pot) were tested for the fitness parameters (see materials and methods for details). See Table S1 for comparative biomass, fruit numbers, and representative proportions of life phases and fruit morphs.

Environment simulation in glasshouse experiments with abiotic and biotic conditions of the optimal habitat of *Ae. arabicum* provided ideal fitness to the plants. Total biomass and the number of fruits produced in a lifetime per plant were significantly higher in control conditions (Ctrl.) than in hydrothermal (HT1-5), nutritional (N1-2) and competitive (C1-3) stress conditions (Figs 2, S2) similar to the sub-optimal edges.

Low proportion of DEH fruit morphs (DEH: IND = 1.7: 1) produced at control optimal habitat conditions is also corroborated by field-collected data (obtained from Çağatay Tavşanoğlu, Hacettepe University, Ankara, Turkey) of the fruit morphs (DEH: IND = 1.9: 1) from the optimal and edge habitats. Approximately 5089 DEH fruits (1089 intact fruits and >20,000 seeds from DEH fruits = 4000 fruits) were collected from optimal habitat compared to 2669 IND fruits in recent field visits in Çankırı district, Inner Anatolian mountain slopes (750–2000 masl).

### A natural stress gradient driven by temperature and moisture differences affected the plasticity of life phases and fruit morphs

**Figure 3:**
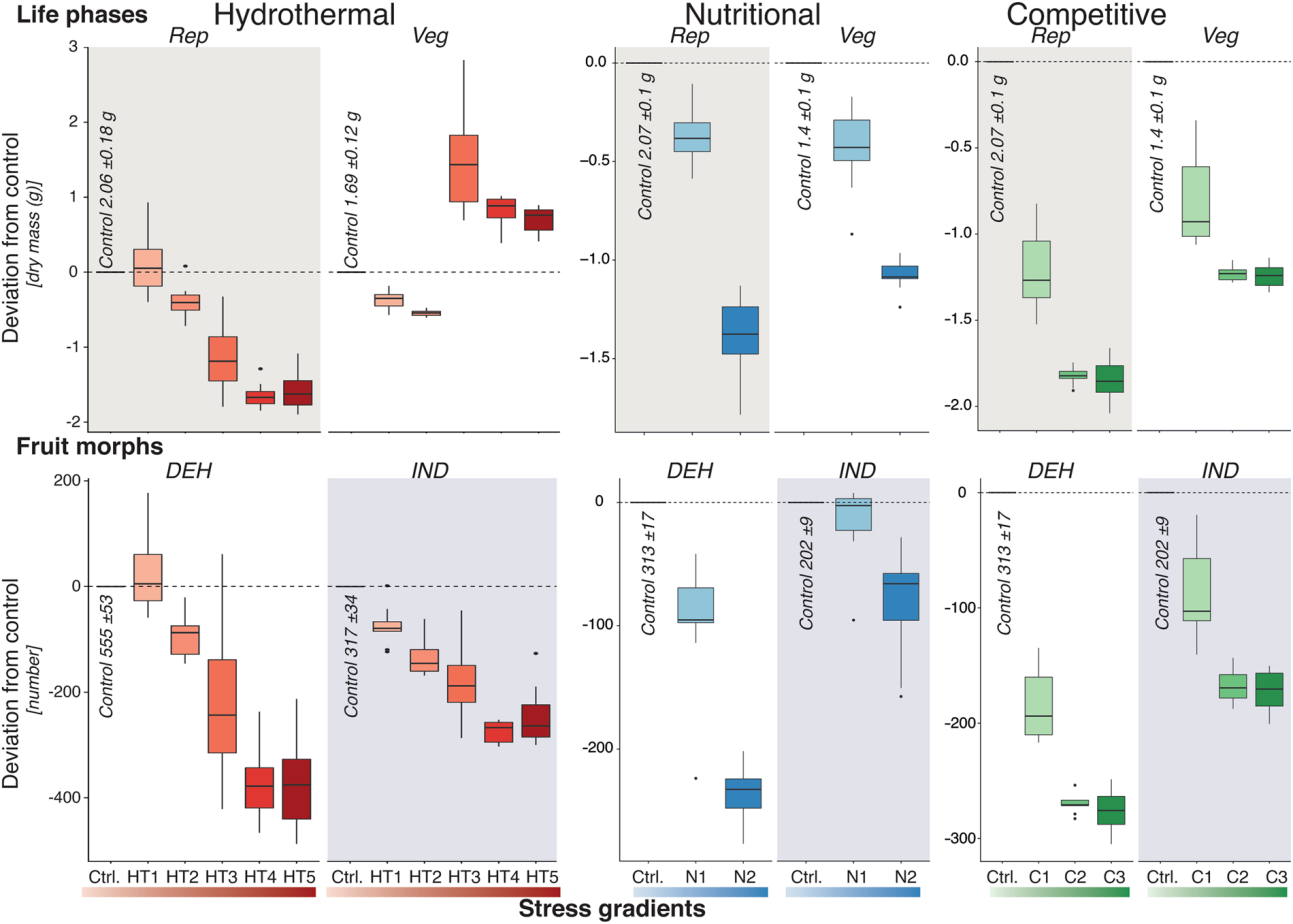
Hydrothermal, nutritional and competitive stress affect plasticity in life phases (vegetative, Veg: reproductive, Rep parts) and fruit morphs (dehiscent, DEH: indehiscent, IND fruits) of *Aethionema arabicum*. Deviation from control (Ctrl.) dry mass (Veg and Rep), and number of fruits (DEH and IND) as an effect of hydrothermal (HT1-5), nutritional (N1, 2) and competitive (C1-3) stress gradients are represented as box and whisker plot. Absolute values of dry mass and number of fruits in each control experiment are mentioned in respective plots. Codes for the stress gradients are explained in Fig. 2.

Hydrothermal stress to the parental generation significantly changed the proportion of vegetative and reproductive parts upon maturity, thus producing a varied number of fruits in their lifetime with a proportional change of DEH vs IND fruits per plant (Fig. 3, Table S3). Similarly, high temperature (thermal stress) and low water availability (drought stress) had significant effects on the plasticity in life phases and fruit morphs (Fig. S3, Table S3). High nutrient deficiency stress, which was equivalent to low available nutrients at high altitude of >2500 msal, had an overall adverse effect on the growth of the plant and produced fewer fruits with less DEH fruit morphs compared to control (Fig. 3, Table S3). Similarly, intraspecific competition did not influence either the vegetative: reproductive parts ratio or the DEH: IND fruit morph ratio (Fig. 3, Table S3).

Biomass is often treated as a fitness proxy and can correlate positively with the reproductive output^42^. We found no significant proportional changes in the dry mass of vegetative and reproductive parts or the number of fruits produced in control and stressed conditions for nutrient and intra-specific competition (Fig. 3, Table S1). These results indicate that the species is capable, although stressfully, to colonize optimal and suboptimal habitats without significant perturbation from sibling competition and from resource availability^12,13^. However, the high proportion (Fig. 3) of multi-seeded DEH fruits generated from high hydrothermal stress conditions produce quick and uniformly germinating DEH seeds with low dormancy and is considered a high-risk strategy. A similar effect of maternal stresses (nutrient, salinity) on seed traits was observed in the dimorphic species, *Suaeda aralocaspica*^43^. Conversely, the single-seeded IND fruit morph, whose seeds show delayed and fractionated germination, offered a low-risk strategy to ensure survival by going into soil seed bank for a long time^29^.

### Thermal and hydrothermal were the major stress gradients influencing plasticity

**Figure 4:**
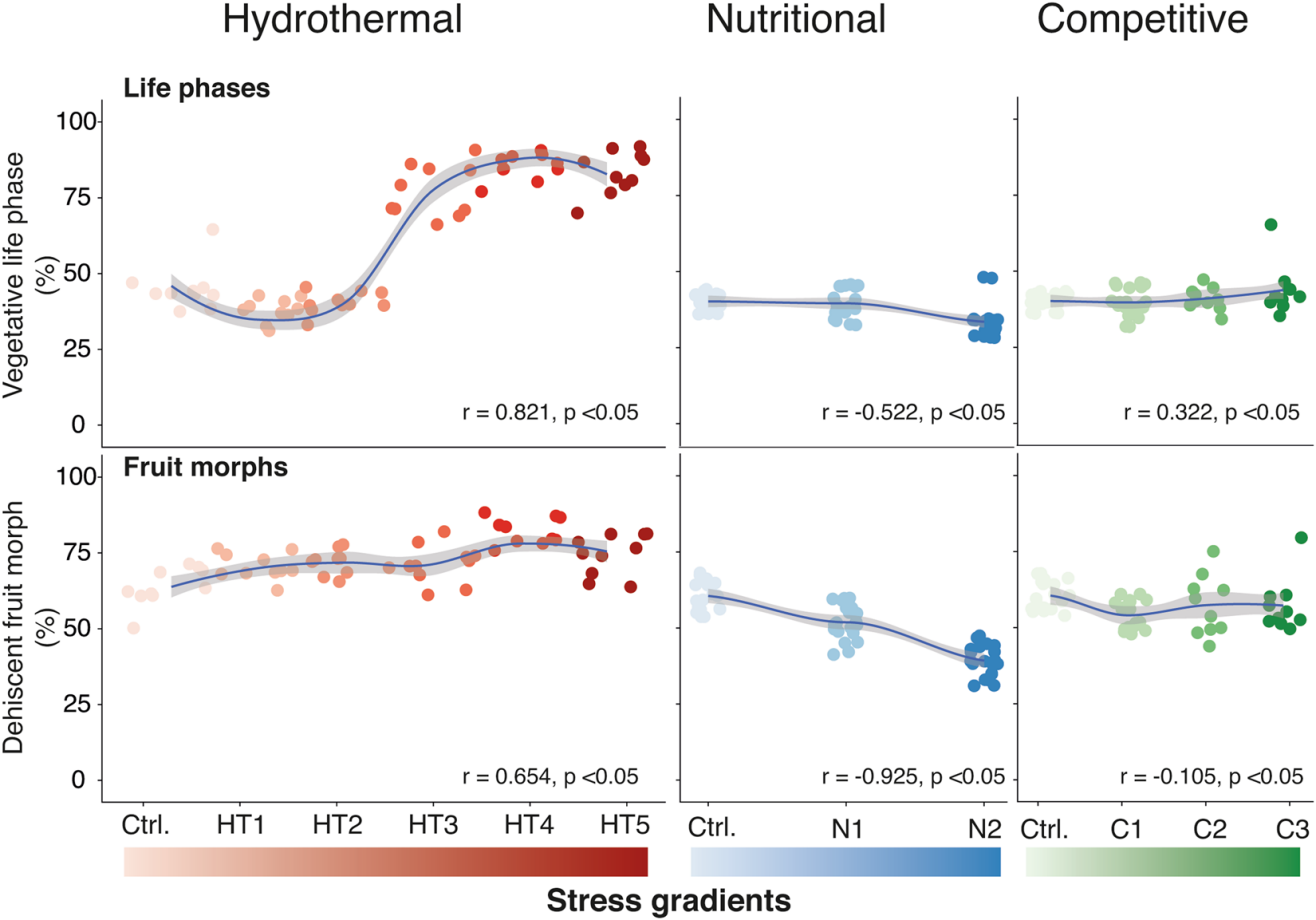
Correlation of hydrothermal, nutritional and competitive stresses on the plasticity in life phase (vegetative: reproductive) and fruit morph (DEH: IND). The blue regression line represent the correlation between the percentage of vegetative life phase (Veg) and dehiscent (DEH) fruit morph, with grey shaded area denoting 95% confidence limit of the regression. Pearson’s correlations are mentioned within each plot. For a comprehensive multivariate canonical correspondence analysis between the stress factors and the plasticity, see Fig. S5 and Tables S4–7).

While plasticity was coherent with the propensity of the stress gradients in the species’ natural habitats, a multivariate analysis revealed the relative influence of simulated stress gradient on the life phase and fruit morph changes (Figs 4, S5). Multivariate canonical correlation analysis (CCA) between the stress and the plasticity responses revealed that thermal and hydrothermal stress uniformly affected both life phase and fruit morph plasticity more than the other stresses (Fig. S5, Tables S4 and 5). The contrasting outcome from our simulated drought stress experiments indicated that the scarcity of water alone could not induce consistent plastic responses as it depends on several stochastic factors along the mountain slopes^41^, whereas an integrated hydrothermal stress gradient could do so. The converse dimensionality of the canonical vectors for the stress factors other than hydrothermal stress also confirmed that a synergistic effect is not evident when thermal, drought, nutritional and competitive stress gradients independently act on life phase or fruit morph ratio (Fig. S5, Table S4).

### Stress-mediated plasticity influences the phenology of the dimorphic *Aethionema arabicum*

**Figure 5:**
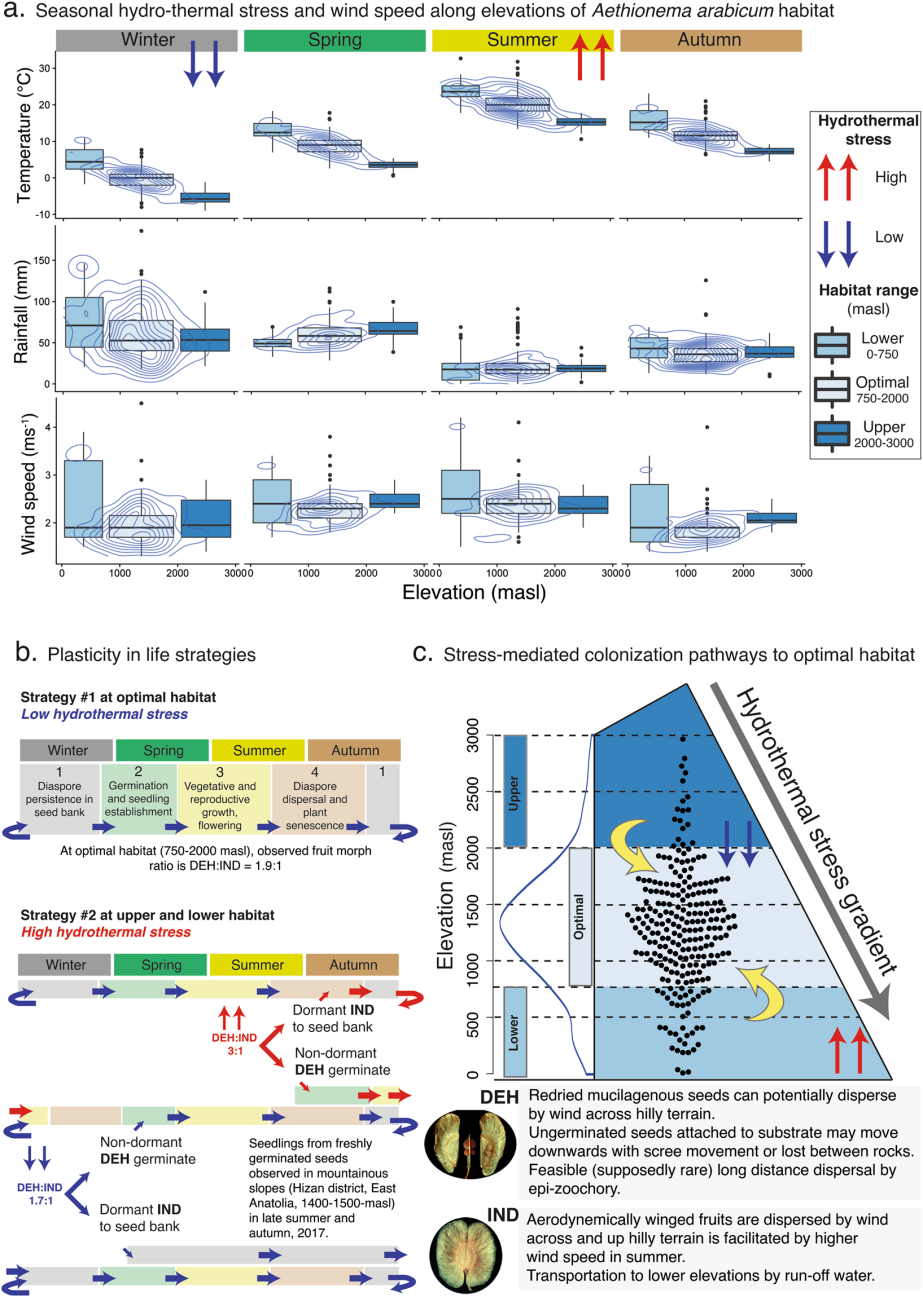
Natural stress gradient (temperature and rainfall) induce flexibility in fruit morph ratio in *Aethionema arabicum* to change their phenology and potentially influence the colonization of optimal habitat with the help of seasonal change in wind speed. (**a)** Seasonal temperature, precipitation and wind speed of the geo-location of 238 accessions of *Ae. arabicum* in ten Mediterranean/Irano-Turanian countries are represented as box and whisker plots. The dimension of more densely concentric contours (blue lines) represents the optimal habitat for the species than the less populated lower and upper habitat ranges computed from their natural occurrence along the elevation. (**b**) The schematic model explains the plasticity in life strategies of *Ae. arabicum* via stress-induced change in phenology and proportion of fruit morphs. The dominant strategy #1 is applicable for optimal habitat with low hydrothermal stress where DEH:IND fruit morph ratio are comparable between experimental results (1.7:1) and field observation (1.9:1). The alternative strategy # 2 is expected at lower and higher altitudes with high hydrothermal stress, resulting in changed fruit morph ratio (3:1) and phenology. Change in the colour of arrows refer to the change in hydrothermal stress conditions (red = high, blue = low). (**c**) The Gaussian density and dot plots of 238 studied *Ae. arabicum* accessions (blue curve) are compared in its optimal habitat (750–2000 m above sea level, masl), higher (2000–3000 masl) and the lower altitudes (0–750 masl). Spatial hydrothermal stress gradients potentially influence the colonisation of optimal habitat via changes in life strategies. Possible pathways of colonization for both fruit morphs (DEH and IND) are briefly explained below the infographic.

Ecological relevance of the stress-mediated plasticity was only apparent with a holistic consideration of the observed natural life cycle of *Ae. arabicum* and seasonal hydrothermal gradient along the habitat (Fig. 5a). In the optimal habitat with low hydrothermal stress, seeds of *Ae. arabicum* germinate from the soil seed-bank during late winter/ early spring (Strategy # 1, stage 1 in Fig. 5b). Subsequently, the seedlings establish themselves, grow into a mature plant (stage 2), produce seeds in dry and hot summer months (stage 3), and the diaspores are dispersed in late summer and autumn (stage 4). Germination timing greatly influences the likelihood of seedling survival and fitness of the progeny as demonstrated in high mountain species, *Primula alpicola* and *Pedicularis fletcheri*, where a synchronized dormancy cycle with the seasonal environmental conditions allowed germination of seeds soon after snowmelt^44^. The inherent difference in diaspore germinability in *Ae. arabicum*, primarily assigned to 34-fold higher ABA content in the IND fruits compared to the seeds from DEH^31^, trigger germination of non-dormant DEH seeds in late summer or autumn immediately after their maturity and dispersal.

In eventual high hydrothermal stress conditions (Strategy #2, Fig. 5b) *Ae. arabicum* plants produce proportionally more DEH fruits (from experimental evidence presented in Figs 3, 4) with immediate seed germination during autumn due to low their dormancy, while the IND fruits remain in the seed bank due to their high dormancy. During recent field studies in 2017 (late summer and early autumn), seedlings from freshly germinated seeds were observed between 1400–1500 masl in the mountainous slopes in Hizan district, East Anatolia (personal communication, M. Firat, Yüzüncüyıl University, Van, Turkey). This immediate germination of DEH seeds shifts the phenology to a subsequent vegetative and reproductive growth phase during cold and wet winter months and experience low hydrothermal stress. This changed phenology conceivably produces a comparably higher proportion of IND fruits (DEH:IND = 1.7:1) than in high-hydrothermal stress conditions (DEH:IND = 3:1), which go into the soil seed bank with their higher dormancy and wait until next spring to germinate and recalibrate to the dominant phenology in strategy #1 (Fig. 5b). This stress-induced shifted phenology can potentially avoid spatial-temporal high stress conditions.

Elevation gradients and associated hydrothermal gradients are considered as discerning factors for intra-and interspecific phenology with contrasting responses in terms of flowering time and seasonal development^45^. In *Capsella bursa-pastoris*, variation in flowering time responses was observed among collection sites at different elevations across Europe^46^. Similarly, low hydrothermal stress in our simulated laboratory experiments produced a fruit morph ratio (DEH: IND = 1.7:1) comparable to the one observed in field studies in 2017 at the optimal habitat (DEH: IND = 1.9:1) and validated our hypothesis on plasticity in life strategies for *Ae arabicum* (Fig. 5b).

### Naturally-regulated plasticity and stress-mediated colonization pathways to optimal habitat

We explained how morphological plasticity, often considered as a mode of adaptation and speciation^4,47^, is relevant to both local adaptation and spatial prevalence for *Ae. arabicum* (Fig. 5c). Both plasticity and phenology shift in response to stresses along the altitudinal distribution of *Ae. arabicum* allowed escape of the diaspores in space and time from the sub-optimal habitats. From field observation and experimental evidence, we suggested possible migration pathways for the diaspores developed under low and high hydrothermal stress conditions to colonize optimal habitat (summarized in Fig. 5c).

Seeds of dehiscent (DEH) fruits developed under low hydrothermal stress or produced in the field at optimal habitat germinated immediately in late summer or early autumn possibly near the mother’s site by anchoring to the substrate with mucilage^31^. However, the possibility of dispersal by rain (ombrohydrochory) cannot be ruled out for both dimorphic diaspores, but it is especially important for the abscission of the IND diaspores^29,31^. Moreover, very rare cases of long-distance transport are possible by adherence to animal vectors (exo-zoochory) as was shown for other Brassicaceae taxa with intercontinental dispersal^48,49^. Likelihood of transport by attaching to the substrates that constantly move downhill in screes at East Mediterranean and Irano-Turanian mountains^28,41^ adds up to the fate of DEH seeds dispersed at uphill elevations to move to the optimal habitat. Indehiscent (IND) fruits developed under similar low-stress conditions remain dormant in the seed bank to germinate later in the spring, follow natural phenology, and maintain the natural fruit ratio at optimal habitat (DEH: IND = 1.7:1).

Consistently high constant wind speed at the low altitudes (2.3 ± 0.6 m/s) with occasional strong wind gusts further enhances the opportunity of IND fruits to be dispersed by anemochory over hilly terrain via their aerodynamically winged pericarp (Fig. 5c). While the mean wind speed (seasonal and spatial) was not significantly different among the habitat ranges, daily maximum wind speed regularly reached more than 5.5 m/s, for several days in a month. Thus, there is a greater probability of experiencing higher wind speed at lower- than at higher- altitudes (representative data of Ankara, 938 masl and Amasia, 405 masl, Turkey, and overview of wind speed and direction map in mountainous regions of Anatolia, Fig. S5). Previous reports about flight capacity of IND fruits in simulated airflow^31^ can be upgraded to a high potential distance by considering the real field conditions (Fig. S5) and the non-diminishing airspeed that enables dispersal from low to high altitudes.

On the other hand, DEH seeds developed under high hydrothermal stress at the lower edge of the habitat shift their reproductive phenology (Fig. 5b) following immediate germination, which was also observed in field visits. The germinated seedlings would eventually experience cold and moist winter during seedling establishment and growth phases to produce a natural fruits ratio (1.7:1). Moreover, the re-dried mucilage of the DEH seeds can help in their dispersal across hilly terrain due to similar aerodynamic properties like winged IND fruits^31^. However, the IND fruits developed under stressed conditions should remain in the seed bank to germinate in the spring to experience again high hydrothermal stress and produce an altered fruit ratio (3:1). Nevertheless, the winged IND fruits can potentially fly upwards with the help of high wind speed (Figs 5a, S5) and occasional strong wind gusts observed in summer in fields at lower edge altitudes.

Altogether, plasticity and a perpetual shift in phenology due to the stress gradients along sub-optimal habitat edges aid adaptive migration and colonization at the optimal habitat (750–2000 masl). Further extensive field experiments on the specific dispersal mode, direction, and proportion of both fruit morphs will elucidate the spatial-temporal dynamics of the species along the mountain slope. Our study indicates that complementary exploration by integrating field observations, ecological modelling, and experimental validation with simulated natural gradients is suitable to test the long-standing hypothesis of adaptive benefits of plasticity in dimorphic species in a tractable way.

## Material and Methods

### Natural distribution of *Aethionema arabicum*

*Aethionema arabicum* is an annual herb widely distributed in the arid and semi-arid environments of East Mediterranean/Irano-Turanian countries (Fig. 1). The geo-location of 238 accessions in 10 countries was determined from field sampling by the working group of Eric Schranz (member of ERA-CAPS “SeedAdapt” consortium project, Wageningen University and Research Centre, Netherlands), private collection and personal observations of Barış Özüdoğru, preserved samples in Hacettepe University Herbarium (HUB) Turkey, JACQ virtual herbaria, Vienna (http://herbarium.univie.ac.at/database), and digitized records in GBIF^50^. An exhaustive compilation of accessions was prepared, which included all possible synonyms of *Ae. arabicum*, from archived documents in Herbaria at Botanical Institute, Azerbaijan (BAK), Department of Plant Taxonomy and Geography, Armenia (ERE), and Georgian National Museum, Georgia (TGM). The accuracy of elevation for the recorded coordinates was validated in www.geoplaner.com. Field data on distribution pattern, fruit morph proportion and natural germination times in the mountainous habitat in Turkey (specifically Inner and South Anatolia) were collected from April to October 2017.

### Climatic data collection

The climate data for the natural habitats of *Ae. arabicum* distributed over 0–3000 m above sea level (masl) were accumulated from the Worldclim database (http://worldclim.org/version2). We used the high spatial resolution GeoTiff images (30′′ grid = ~1 km^2^) to extract the average monthly climate data for minimum, mean, and maximum temperature (°C), rainfall (mm) and wind speed (m s^−1^) for 1970–2000 in R (ver. 3.4.3) using the packages raster, sp, rgdal, foreach, ggmap, rJava and xlsx^51^.

### Edaphic data collection

All available soil parameters for 238 accessions were collected from global soil grid database^52–54^ (https://soilgrids.org). Data for soil coarse fragment percentage (v/v), pH in H_2_O, cation exchange capacity (cmol + kg^−1^), and soil organic carbon content (g kg^−1^) at 0.05 m depth from the surface were extracted from GeoTiff images (resolution 1 km^2^) in R.

### Seed collection and germination

Mature plants were grown from seeds originally collected from Turkey in 2007 and provided by Eric Schranz^29^. After-ripened seeds were stratified on sterilized rooting-media agar plates (0.043% Murashig & Skoog Medium basal salt mixture, Duchefa, Haarlem, Netherlands; 1% Agar; pH 7) for four days at 4 °C in darkness followed by incubation in a growth chamber at 14 °C with 16 h daylight (155 µm s^−1^ m^−2^). After 15–20 days, the germinated seedlings were transferred to 0.5 l pots filled with soil consisting of Einheitserde (Einheitserdewerke Gebr. Patzer GmbH & Co. KG): autoclaved sand: perlite (7:2:1). The plants started flowering one month after germination and produced seeds after another two months.

### Controlled growth conditions

Environmental conditions of the optimal habitat with high species distribution (750–2000 masl) were considered optimal for the growth of *Ae. arabicum* and used as control parameters, along with parameters extracted from previous hydrothermal modelling of seed germination and growth^29^. All control experiments (Ctrl.) were conducted in long-day light condition (16 h light) and optimal temperature cycle (20/12 °C, day/night), corresponding to the natural phenology (Fig. S1a) of the species in spring and summer^55^ in East Mediterranean/Irano-Turanian countries. Each group of 15 plants received 800 ml of water (≡55 mm rainfall) and ca. 35 ml of added fertilizer (0, 2% v/v solution of Wuxal Super, Aglukon, Düsseldorf, Germany) every week.

### Induced stress conditions

#### Hydrothermal stress

Hydrothermal stress gradients on the life phase and fruit morph plasticity were simulated in laboratory by providing 600 ml (HT1 ≡ 35 mm rainfall, 20/12 °C) and 400 ml (HT2 ≡ 20 mm rainfall, 20/12 °C) water, respectively to batches of 15 plants per week for each treatment. High thermal stress was induced at four-leaf rosette stage by exposing batches of 15 plants to higher temperature (HT3, 25/20 °C). To test the natural equivalent of hydrothermal stress on plasticity, two more binary combinations of stress gradients (HT4, 35 mm, 25/20 °C; HT5, 20 mm, 25/20 °C) were examined on groups of 15 plants.

#### Nutritional stress

As suggested by the altitudinal gradient of soil cation exchange, pH and organic carbon content, equivalent nutrient stresses were tested by supplying less (N1, 0,1% v/v, moderate stress) or no additional nutrient (N2, water placebo, high stress) contrary to the control nutrient supply (0.2% v/v).

#### Competitive stress

The observed altitudinal gradient of soil coarse fragment size, which potentially regulates the habitability of plants in a particular habitat, were extrapolated to test the intra-species competitive stress. Instead of only one plant per 0.5 l pot in control experiments (no stress), three (C1, low stress), five (C2, moderate stress) or seven plants (C3, high stress) were grown together with identical growth conditions. The target plant was surrounded by competitor plants during growth (Fig. S6).

### Data collection for life phase and fruit morph plasticity after stress treatments

After 3 months of stress treatments, aboveground vegetative parts (stem and leaves) and reproductive parts (fruit valves; dehiscent, M + seeds; and indehiscent, M- seeds) from each of the treated and control plants were collected individually in paper bags for drying in an oven at 80 °C for 48 h and weighed for dry mass. Roots were excluded from the measurement of vegetative structure in all cases due to the technical difficulties of washing off soil/sand particles without significantly damaging the root system. The number of DEH and IND fruits was counted for each control and treated plant.

### Statistical analysis

The average monthly data for climatic parameters were aggregated for the four seasons as follows: winter (Dec, Jan, Feb), spring (Mar, Apr, May), summer (Jun, Jul, Aug), autumn (Sep, Oct, Nov). All climatic and edaphic factor data were subjected to Locally Weighted Least Squares Regression (Loess) in R with the respective elevation at geo-locations of accessions with a 2D polar contour analysis to determine the optimal habitat features. Depending on the Gaussian distribution of *Ae. arabicum* along the elevation, the habitat range was further categorized into lower (0–750 masl) and upper edge (2000–3000 masl) of the optimal habitat (750–2000 masl).

Analysis of variance (ANOVA) was performed to compare the effect between control and stress treatment conditions on the dry mass of vegetative- (Veg.) and reproductive- (Rep.) plant parts and proportion of dehiscent (DEH) to indehiscent (IND) fruits, followed by post-hoc mean grouping with SPSS (version 24). For all non-parametric and proportional data, Kruskal-Wallis test (H) was performed to compare the effect of the individual or combined stress levels on life phase and fruit morph plasticity. A significant difference of means from the control was determined by Dunn’s pairwise tests for H and adjusted using the Bonferroni correction in SigmaPlot 13.0.

To determine the effective magnitude of the stress gradient to the plasticity and interactive correspondence between the life phase and fruit morph changes we performed multivariate canonical correlation analysis in R package ‘cca’ and ‘vegan’.

## Supplementary information

Supplemental information

## Data Availability

All data generated or analysed during this study are included in this published article (and its Supplementary Information Files). Datasets that are more detailed are available from the corresponding author on reasonable request.
