# Supplementary material for: Naturally-primed life strategy plasticity of dimorphic Aethionema arabicum facilitates optimal habitat colonization
Source: Sci Rep. 2019 Nov 6;9:16108. doi: 10.1038/s41598-019-52520-y (PMC6834856; doi:10.1038/s41598-019-52520-y)
Supplement: Supplementary file 1 — Supplemental information [file 41598_2019_52520_MOESM1_ESM.pdf]

<u>Supporting Information</u>

Naturally-primed life strategy plasticity of dimorphic *Aethionema arabicum* facilitates optimal habitat colonization

Samik Bhattacharya, Katja Sperber, Barış Özüdoğru, Gerhard Leubner-Metzger, Klaus Mummenhoff

The following Supporting Information is available for this article:

Fig. S1. Climatic and edaphic factors along the altitudinal range of *Aethionema arabicum.*

Figure S2. Comparative phenotype of control and stress-induced (thermal and competitive) plants.

Fig. S3. Effect of thermal and drought stress on plasticity in life phases and fruit morphs of *Aethionema arabicum*.

Fig. S4. Multivariate correlation analysis revealed thermal and hydro-thermal stresses influencing the plasticity in life phase and fruit morph more than the other abiotic and biotic stresses.

Fig. S5. Representative monthly variation in wind speed and direction in the Mediterranean/ Irano-Turanian region.

Fig. S6. Intra-species competitive stress experimental design.

Table S1. Seasonal temperature, rainfall and wind speed at optimal, lower and upper habitats of *Aethionema arabicum*

Table S2. Comparative dry mass of vegetative and reproductive parts and number of dehiscent (DEH) and indehiscent (IND) fruits between control and stress experiments.

Table S3. One way ANOVA table for plasticity in life phases (vegetative: reproductive) and fruit morphs (DEH: IND) in response to stress gradients in *Aethionema arabicum*

Table S4. Tests of Canonical Dimensions for stress gradient vs plasticity responses

Table S5. Standardized Canonical Coefficients for stress gradient and plasticity responses

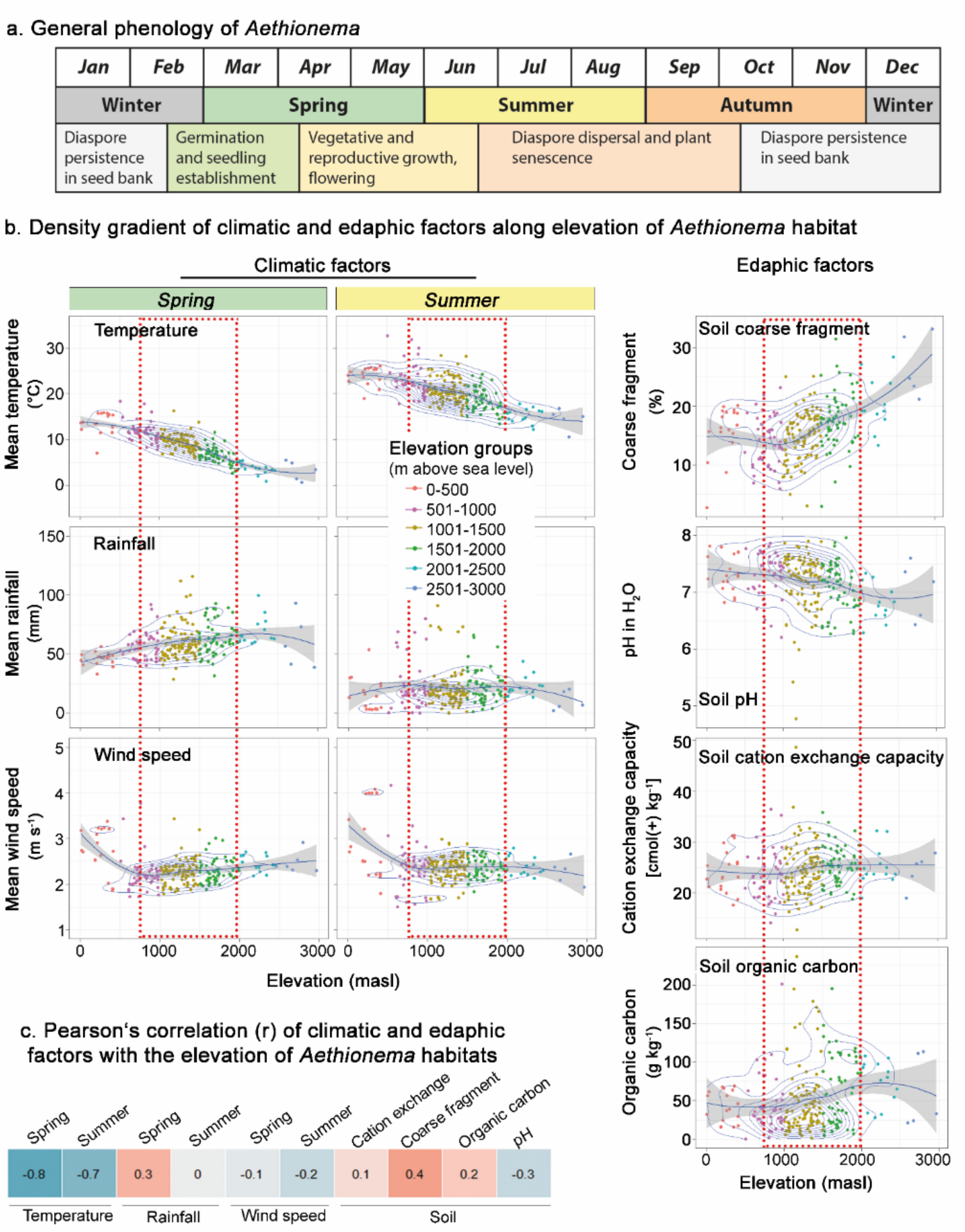

a. General phenology of Aethionema
Jan
Feb
Mar
Apr
May
Jun
Jul
Aug
Sep
Oct
Nov
Dec
Winter
Spring
Summer
Autumn
Winter
Diaspore persistence in seed bank
Germination and seedling establishment
Vegetative and reproductive growth, flowering
Diaspore dispersal and plant senescence
Diaspore persistence in seed bank
b. Density gradient of climatic and edaphic factors along elevation of Aethionema habitat
Climatic factors
Edaphic factors
Spring
Summer
Temperature
Mean temperature (°C)
Rainfall
Mean rainfall (mm)
Wind speed
Mean wind speed (m s-1)
Elevation groups (m above sea level)
0-500
501-1000
1001-1500
1501-2000
2001-2500
2501-3000
Elevation (masl)
Soil coarse fragment
Coarse fragment (%)
Soil pH
pH in H2O
Soil cation exchange capacity
Cation exchange capacity [cmol(+) kg-1]
Soil organic carbon
Organic carbon (g kg-1)
Elevation (masl)
c. Pearson's correlation (r) of climatic and edaphic factors with the elevation of Aethionema habitats
Spring
Summer
Spring
Summer
Spring
Summer
Cation exchange
Coarse fragment
Organic carbon
pH
-0.8
-0.7
0.3
0
-0.1
-0.2
0.1
0.4
0.2
-0.3
Temperature
Rainfall
Wind speed
Soil

Figure S1. Climatic and edaphic factors along the altitudinal range of *Aethionema arabicum* facilitate their colonization to its optimal habitat (750-2000 masl) as compared to its lower (<750 masl) and upper (> 2000 masl) edges. a. General phenology of *Ae. arabicum* highlights the germination of seeds during spring from the soil seed bank, followed by vegetative and reproductive growth throughout summer, and persistence of diaspores in the soil seed bank during winter. **b.** Correlations of climatic factors during spring and summer with the habitat are presented as contour curves of species density along the elevation. Densely concentric contours defines the optimal habitat for the species and are marked with red dotted rectangles (750-2000 masl), with the lower (0-750 masl) and upper (2000-3000 masl) habitat edges. The average seasonal climate data temperature (°C), precipitation (mm), wind speed (ms^{-1}) for 1970-2000 were extracted from Worldclim-global climate database (http://worldclim.org/version2). The average monthly data for all climatic parameters were first aggregated for the four seasons consistent with the distribution of the accessions: winter (Dec, Jan, Feb), spring (Mar, Apr, May), summer (Jun, Jul, Aug), autumn (Sep, Oct, Nov). All edaphic factors were extracted from global soil grid database (https://soilgrids.org). **c.** Pearson's correlation values (r) of the climatic and edaphic factors with the elevation of the species distribution were presented as gradient colors (blue= negative, red= positive, grey= no correlation).

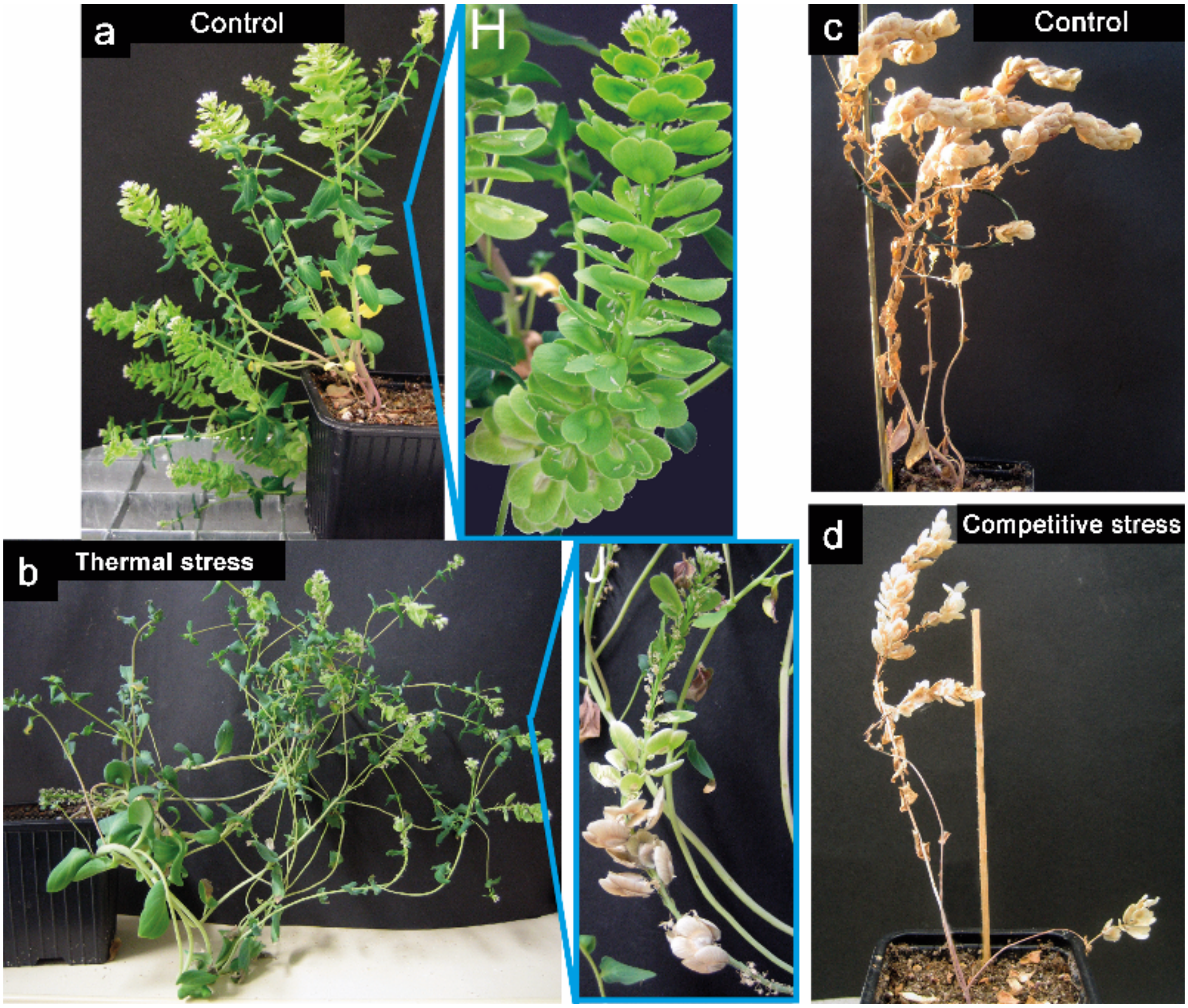

Figure S2. Comparative phenotype of control and stress-induced (thermal and competitive) plants. Plants kept in controlled condition visibly produce more reproductive parts and more fruits (**a**) compared to the plants under thermal stress producing long branches and fewer fruits (**b**). Intra-specific competition (**d**) significantly reduced the biomass (Δg = 1.94 to 3.1 g) and the total number of fruits produced (Δn = 274 to 448) than the plants grown alone in an isolated pot (**c**), but did not affect the proportion of either vegetative: reproductive parts or DEH: IND fruit morphs.

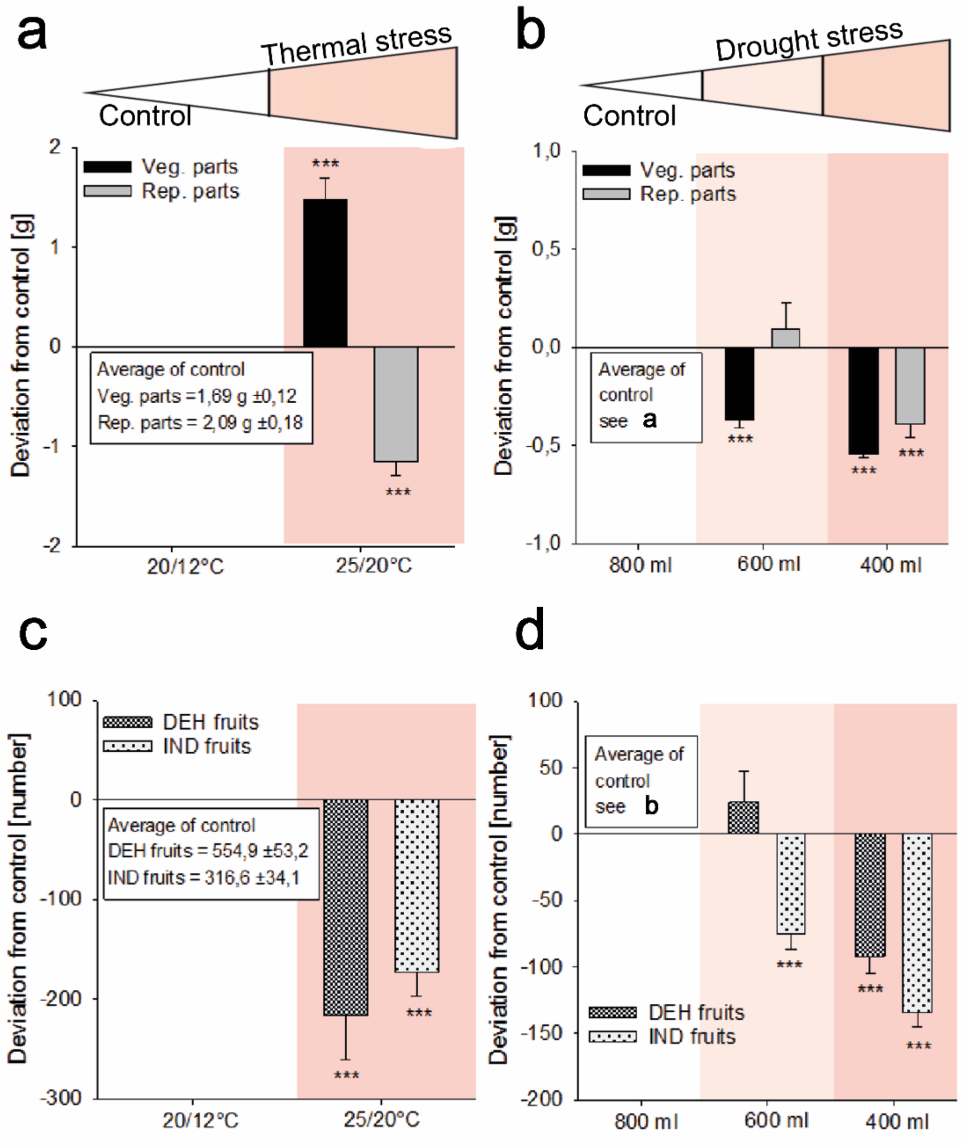

Fig. S3. Effect of thermal and drought stress on plasticity in life phases (vegetative, Veg.; reproductive, Rep. parts) and fruit morphs (dehiscent, DEH; indehiscent, IND fruits) of *Aethionema arabicum*. Mean (± SE) deviation from the control dry mass or number of fruits of each morph (mentioned within box in each plot) are represented as bar plot. Analysis of variance (ANOVA) was performed to compare the effect between thermal stress (25/20 °C, 16/8 h light: dark cycle) and controlled growth (20/12°C) on the dry mass of vegetative- (Veg.) and reproductive- (Rep.) plant parts (**a**) and fruit morphs (DEH and IND; **c**). Kruskal-Wallis test (H) or ANOVA was performed to compare the effect of the stress levels on life phase **(b)** and fruit morph **(d)** plasticity. A significant difference of means from control (*** = p<0.001) were determined by Dunn's pairwise tests for H (P < 0.01, adjusted using the Bonferroni correction) or Tukey's multiple range test (P < 0.01) of an ANOVA.

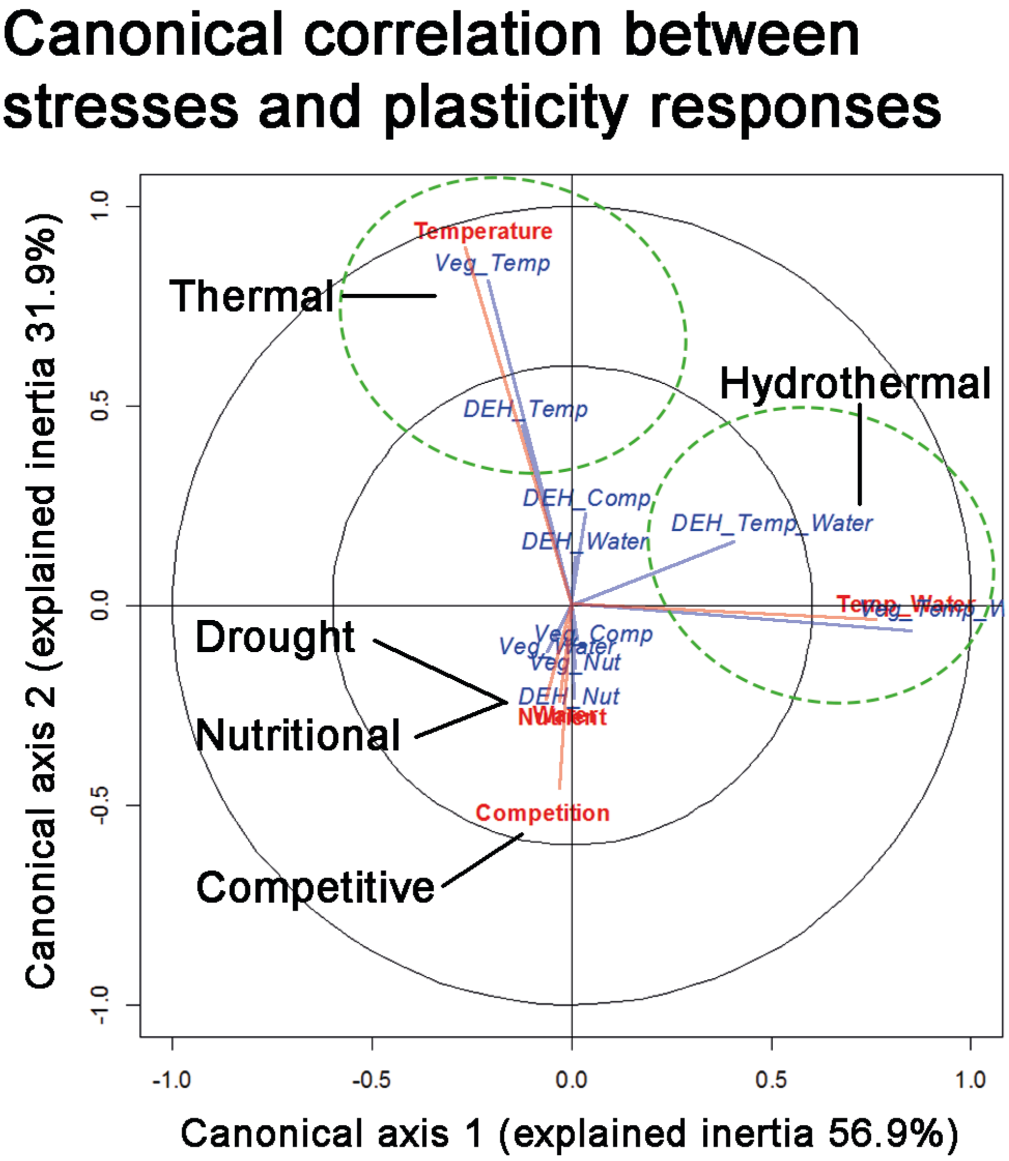

Fig. S4. Multivariate correlation analysis revealed thermal and hydro-thermal stresses influencing the plasticity in life phase (vegetative: reproductive) and fruit morph (DEH: IND) more than the other abiotic and biotic stresses. The canonical correlation between the stress gradient and the plasticity responses are represented as biplots for the first two canonical axes explaining the canonical inertia of stress gradient (red lines) with their effect on the plasticity of life phases and fruit morphs (blue lines). The green dotted circle denoted the grouped gradient with resolving unidirectional canonical dimensions signifying their correlation. (See Tables S4, 5 for canonical dimensions).

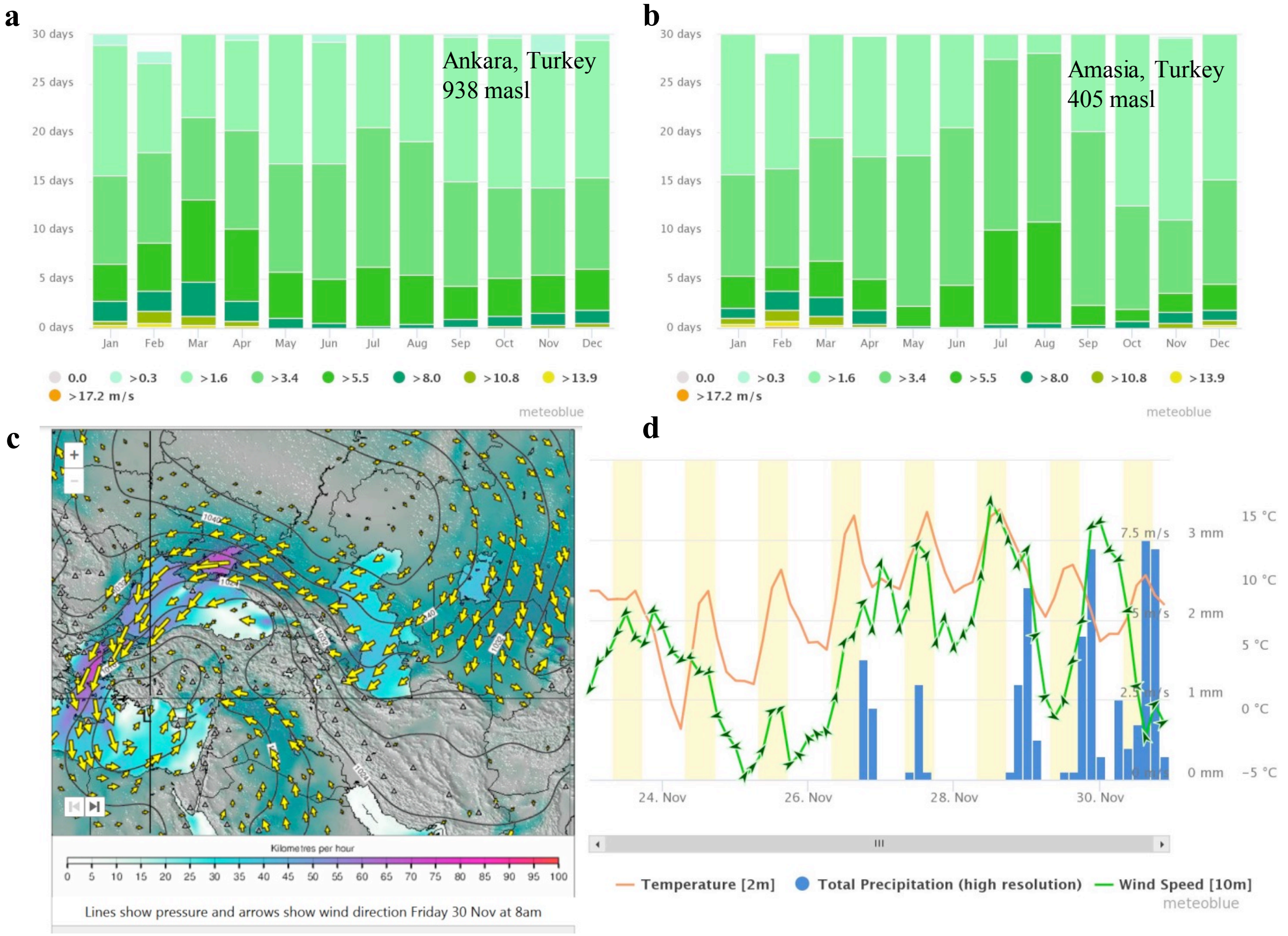

Fig. S5. Representative monthly variation in wind speed and direction in the Mediterranean/ Irano-Turanian region. Monthly wind speed variation for Ankara (**a,** 938 masl, Central Anatolia), and Amasya (**b,** 405 masl, Northern Anatolia) suggest several days with high wind speed than the seasonal mean wind speeds (in Fig. 5a, Table S2). **c.** Representative wind speed and direction topology data (30th November, 2018) in the Mediterranean/ Irano-Turanian mountains exhibit low wind speed at higher elevations. **d.** variation in wind speed and direction within a week (24th – 30th November, 2018) at Amasia, Turkey highlight occasional high wind gust. All data were accessed at www.meteoblue.com on 30th November, 2018.

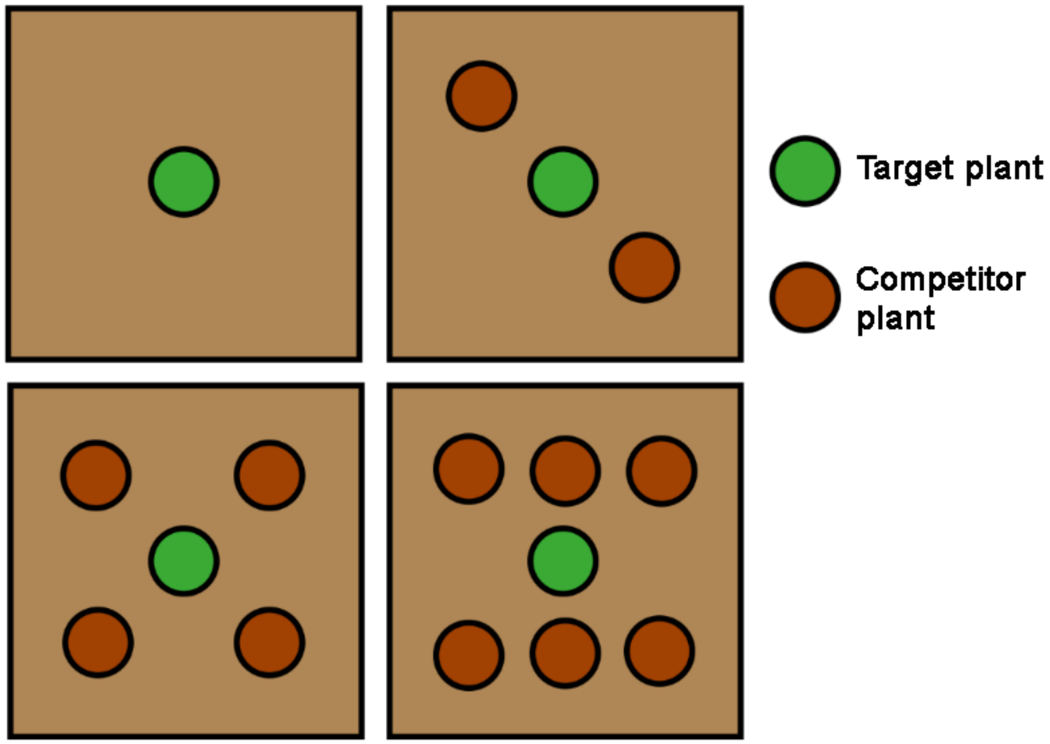

Fig. S6. Intra-species competitive stress experimental design. Only one target plant (green circle) per 0.5 l pot represent control experiments (no stress). Three (low stress), five (moderate stress) or seven plants (high stress) were grown together with target plants at the center surrounded by the competitor plants (brown circles) during growth. Fitness measurements were taken only from the target plant.

Table S1. Seasonal temperature, rainfall and wind speed at optimal, lower and higher habitats of *Aethionema arabicum*

		Temperature (°C)					Rainfall (mm)					Wind (m s^{-1})				
	Elevation	Mean	Winter	Spring	Summer	Autumn	Mean	Winter	Spring	Summer	Autumn	Mean	Winter	Spring	Summer	Autumn
**Optimal habitat (750-2000 masl)**																
Min	755	4.4	-8	2.6	13.4	6.4	19.4	18.7	28.7	0	12	1.6	1.3	1.8	1.6	1.4
1st Qu	1101	8.55	-2	7.15	18.3	10.3	35.45	40.5	51.3	12.5	27	2	1.7	2.1	2.2	1.7
Median	1347	10.2	0	9	20	11.6	44	52.7	58.3	17.3	36	2.1	1.9	2.3	2.4	1.9
Mean	1344	10.08	-0.47	8.8	20.29	11.67	45.12	61	61.25	21.55	36.62	2.10	1.92	2.28	2.37	1.86
3rd Qu	1585	11.2	1	10.3	21.8	12.65	52.1	77	68	24.85	43	2.2	2.15	2.4	2.5	1.9
Max	1990	19.6	7.7	17.8	31.8	21	108.1	185.7	116	91	125.7	4.1	4.5	3.8	4.1	4
**Leading edge (2000-3000 masl)**																
Min	2016	2.1	-9	0.6	10.6	4.5	19.1	21.7	38.7	2	9.3	1.9	1.4	2.2	1.9	1.8
1st Qu	2181	4.6	-6.6	2.9	14.43	6.575	40.5	39.95	60.48	15.15	32.4	2.1	1.7	2.325	2.2	2
Median	2334	5	-5.8	3.55	15.25	7.15	44.85	53.5	64.35	18.8	36.85	2.25	1.95	2.4	2.3	2.05
Mean	2370	5.06	-5.34	3.43	15.02	7.12	45.01	55.71	67.28	19.44	37.56	2.26	2.08	2.47	2.36	2.09
3rd Qu	2558	5.83	-4.2	4.275	16.18	8.075	52.58	66.58	74.72	22.5	45.05	2.4	2.475	2.6	2.55	2.2
Max	2963	7.2	-1.2	5.4	17.8	9.2	64.9	111.7	99.7	44	62	2.7	2.9	2.9	2.8	2.5
**Rear edge (0-750 masl)**																
Min	8	8.8	-1.7	7	17.8	11	20	21	33	0.3	13.3	1.5	1.5	1.7	1.5	1.4
1st Qu	265	12.2	2.4	11.5	22.2	13.1	37.8	44.7	45.3	4.7	31.7	1.9	1.7	2	2.2	1.6
Median	408	13.9	4.4	12.4	23.6	15.2	47.6	71	49	17.7	43	2.3	1.9	2.4	2.5	2.3
Mean	448.4	14.38	5.25	12.92	23.64	15.7	47.89	76.94	50.09	21.48	43.05	2.4	2.33	2.45	2.66	2.12
3rd Qu	671	16.9	7.7	14.9	25.2	18.4	55.8	105	54	25	56	2.9	3.3	2.9	3.1	2.8
Max	737	20.6	10.9	18.3	32.7	23.1	64.3	146.3	69.3	69	68.7	3.6	3.9	3.4	4.2	3.4

Table S2. Comparative dry mass (g, mean±SE) of vegetative (Veg.) and reproductive (Rep.) parts and number of dehiscent (DEH) and indehiscent (IND) fruits between control experiments and thermal, drought, hydro-thermal, nutrient and competitive stress experiments.

	Control		Thermal stress		Drought stress				Hydro-thermal stress			
			25/20 °C		600 ml		400 ml		25/20 °C+ 600 ml		25/20 °C+ 400 ml	
Veg. parts[1]	1.69±0.12	45.1%[3]	3.17±0.21	77.4%[3]	1.32±0.04	38%[3]	1,15±0,01	40,5%[3]	2,51±0,06	85,4%[3]	2,39±0,05	83,4%[3]
Rep. parts[1]	2.09±0.18		0.94±0.14		2.19±0.13		1.7±0.07		0.44±0.05		0.49±0.08	
Total DM[1]	3.78±0.24		4.11±0.28		3.51±0.15		2.84±0.07		2.96±0.1		2.88±0.1	
DEH fruits[2]	554.9±53.2	63.7%[4]	338.6±44.6	71.3%[4]	579.1±23.2	70.5%[4]	462,8±12,9	71,8%[4]	180±21,8	81,9%[4]	179,1±27,8	74,5%[4]
IND fruits[2]	316.6±34.1		143.3±23.7		241.6±11.3		182.2±10.7		41.9±6.6		60.5±17.6	
Total NoF[2]	871.5±82.5		481.9±65.8		820.7±23.7		645±16.7		222.1±27.9		239.6±44.6	

	Control		Nutritional stress				Competitive stress					
			0,1%		0%		3 plants per pot		5 plants per pot		7 plants per pot	
Veg. parts[1]	1.4±0.09	40.2%[3]	1.04±0.08	39.6%[3]	0.33±0.03	33.5%[3]	0,59±0,09	39,8%[3]	0,17±0,01	41%[3]	0,16±0,02	43,8%[3]
Rep. parts[1]	2.07±0.1		1.6±0.12		0.67±0.07		0.86±0.08		0.25±0.02		0.23±0.04	
Total DM[1]	3.48±0.18		2.64±0.18		1±0.09		1.45±0.16		0.42±0.03		0.38±0.06	
DEH fruits[2]	312.8±16.5	60.5%[4]	216.2±16.1	51.9%[4]	75.9±7.4	39.3%[4]	128,5±9,5	54,1%[4]	42,8±2,4	57,4%[4]	36,6±5,8	57,3%[4]
IND fruits[2]	202.4±9.1		198.8±15.5		121.5±13.6		112.8±12.9		33.9±4.6		30.6±5.6	
Total NoF[2]	515.2±20		415±27		197.4±20.2		241.3±21.9		76.6±6.1		67.2±11.2	

[1] Total dry mass (Total DM), vegetative (Veg. parts) and reproductive parts (Rep. parts) in g
[2] Total number of fruits (Total NoF), dehiscent (DEH fruits) and indehiscent fruits (IND fruits) in number of fruits
[3] Percentages of vegetative parts
[4] Percentages of dehiscent fruit

Table S3. One way ANOVA table for plasticity in life phases (vegetative: reproductive) and fruit morphs (DEH: IND) in response to stress gradients in *Aethionema arabicum*

Source		life phases (vegetative: reproductive)		fruit morphs (DEH: IND)	
	df	***F***	***P***	***F***	***P***
**Thermal**					
Between groups	1	88.87	<0.001	6.82	<0.001
Within groups	18				
**Drought**					
Between groups	2	5.12	<0.05	7.38	<0.005
Within groups	27				
**Hydrothermal**					
Between groups	5	137.12	<0.001	11.18	<0.05
Within groups	54				
**Nutrient**					
Between groups	2	3.24	0.055	39.32	<0.005
Within groups	27				
**Competition**					
Between groups	3	1.22	0.317	1.35	0.272
Within groups	36				

Table S4: Tests of Canonical Dimensions for stress gradient vs plasticity responses

Axis	*Canonical correlation*	*Wilks Lambda*	*F*	*df1*	*df2*	*P*
1	0.98	0.00	19.50	50.00	208.60	1.52E-55
2	0.95	0.01	11.88	36.00	174.12	5.96E-31
3	0.91	0.09	7.34	24.00	136.92	5.09E-15
4	0.64	0.51	2.77	14.00	96.00	1.74E-03
5	0.37	0.86	1.31	6.00	49.00	2.72E-01

Table S5: Standardized Canonical Coefficients for stress gradient and plasticity responses

Variables	Axes		
	1	2	3
Stress gradient			
Thermal	-0.22088	0.939088	-0.25375
Drought	-0.01807	-0.26872	-0.96057
Hydro-thermal	0.832102	0.004051	0.495814
Nutritional	-0.02365	-0.27295	-0.95219
Competitive	-0.07332	-0.51961	0.398608
Plasticity response to stress			
Veg x Thermal	-0.20537	0.905529	-0.25538
DEH x Thermal	-0.11808	0.520665	-0.14684
Veg x Drought	-0.04109	-0.10928	-0.24437
DEH x Drought	0.031679	0.170739	0.538018
Veg x Hydro-thermal	0.975885	-0.00993	-0.01895
DEH x Hydro-thermal	0.51235	0.218155	0.237227
Veg x Nutritional	0.005331	-0.15224	-0.641
DEH x Nutritional	-0.01154	-0.24081	-0.91801
Veg x Competitive	0.051399	-0.07503	-0.10448
DEH x Competitive	0.040143	0.28362	-0.31006
